# Optimization of a bioelectrochemical system for 2,4-dichloronitrobenzene transformation using response surface methodology

**DOI:** 10.1039/c8ra10110h

**Published:** 2019-01-18

**Authors:** Hui Chen, Donghui Lu, Caiqin Wang, Linlin Chen, Xiangyang Xu, Liang Zhu

**Affiliations:** Institute of Environment Pollution Control and Treatment, Department of Environmental Engineering, Zhejiang University Hangzhou 310058 China felix79cn@hotmail.com +86 571 88982343 +86 571 88982343; Zhejiang Province Key Laboratory for Water Pollution Control and Environmental Safety Hangzhou 310058 China; Zhejiang Provincial Key Laboratory of Water Pollution Control 388 Yuhangtang Road Hangzhou 310058 China

## Abstract

In the present study, a bioelectrochemical system (BES) was developed for 2,4-dichloronitrobenzene (DClNB) transformation. Response surface methodology (RSM) was applied to optimize the operational conditions, including the *V*/*S* ratio (volume of the BES/size of the electrode ratio), interval (*D*) (distance between the anode and cathode) and position (*P*) (proportion of the electrodes immerged in the sludge). The optimum conditions for the *V*/*S* ratio, interval and position were 40, 2.31 cm and 0.42. The pollutant removal rate and increase in Cl^−^ were 1.819 ± 0.037 mg L^−1^ h^−1^ and 11.894 ± 0.180 mg L^−1^, which were close to the predicted values (1.908 mg L^−1^ h^−1^ and 12.485 mg L^−1^). A continuous experiment indicated that the pollutant removal efficiency in the BES with 50% of the electrodes immerged in the sludge was 34.6% and 22.6% higher than that in the ones with 0 and 100% of the electrodes immerged in the sludge.

## Abstract 2

In the present study, a bioelectrochemical system (BES) was developed for 2,4-dichloronitrobenzene (DClNB) transformation.

## Graphical abstract

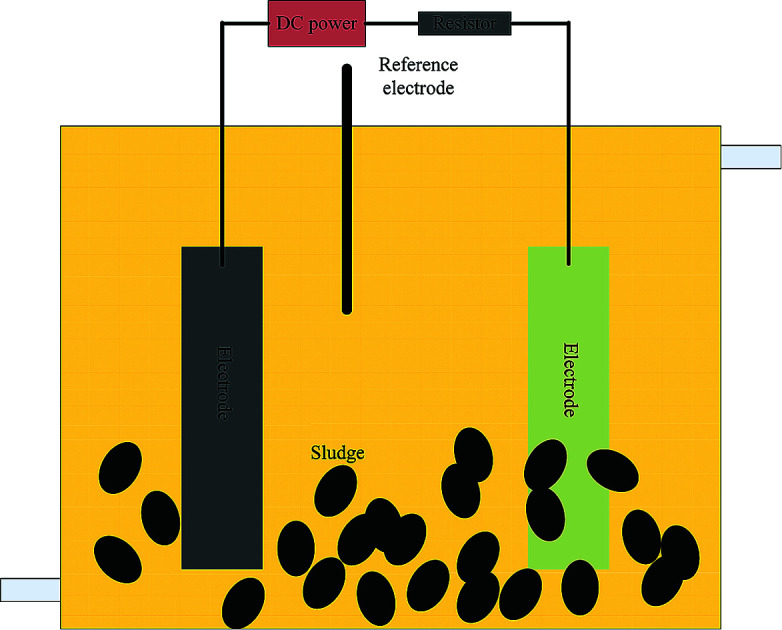

## 1 Introduction

Chloronitrobenzenes (ClNBs), a kind of important raw material used in the pharmaceutical, dye and pesticide industries, are toxic compounds with mutagenic, carcinogenic and teratogenic effects.^1,2^ They pose a serious threat to human beings and livestock by causing liver disease, hemolytic anemia, *etc.*^3^

Bioelectrochemical conversion, which combines biodegradation with electrochemical reduction, has been proven to be an alternative method for contaminant detoxification in recent years.^4^ Bioelectrochemical systems (BESs) are innovative and energy saving compared with the conventional anaerobic and electrochemical processes. This technology has been successfully used in the degradation of substituted aromatic compounds, *e.g.*, azo dyes, chloroethenes, chloronitrobenzenes (ClNBs), *etc.*^5–9^ Extracellular electron transfer related genes which may be responsible for enhanced organohalide-respiration and cathode-respiration activities could be enriched in BESs, contributing to aromatic compound degradation.^10^ Our previous studies confirmed the feasibility of a coupled bioelectrochemical process for the treatment of ClNB-containing wastewater. The 4-ClNB and 2,4-DClNB removal efficiencies in the coupled system were much higher than those of the control; meanwhile, dechlorination-related microbes were enriched in the presence of an external voltage.^1,11^ Recently, Sun *et al.* have investigated the effects of some key parameters on azo dye reduction, including initial pollutant concentration, applied voltage and co-substrates.^12^ In another study treating 2,4-dinitrochlorobenzene using a BES, the effects of voltage, hydraulic retention time (HRT) and salinity were investigated.^13^ However, studies on the optimization of the electrochemical parameters in a system for treating ClNB-containing wastewater are limited, especially related to the optimization of the electrode-related parameters. Response surface methodology (RSM), a set of mathematical techniques describing the relation between independent variables and responses, was developed by Box and Wilson in the 1950s.^14,15^ Nowadays, RSM has been widely used for designing experimental models and determining the optimum experimental conditions.^16–19^

In this study, the objective was to characterize the main parameters in the bioelectrochemical process, including the *V*/*S* ratio (volume of the BES/size of the electrode ratio), interval (*D*) (distance between the anode and cathode) and position (*P*) (proportion of the electrodes immerged in the sludge). The experiments were conducted in a batch assay to optimize the *V*/*S* ratio, interval and position to achieve the best performance in pollutant transformation. The evaluation was conducted with central composite design (CCD), a common type of RSM.

## 2 Materials and methods

### 2.1 Experimental set-up

**Fig. 1:**
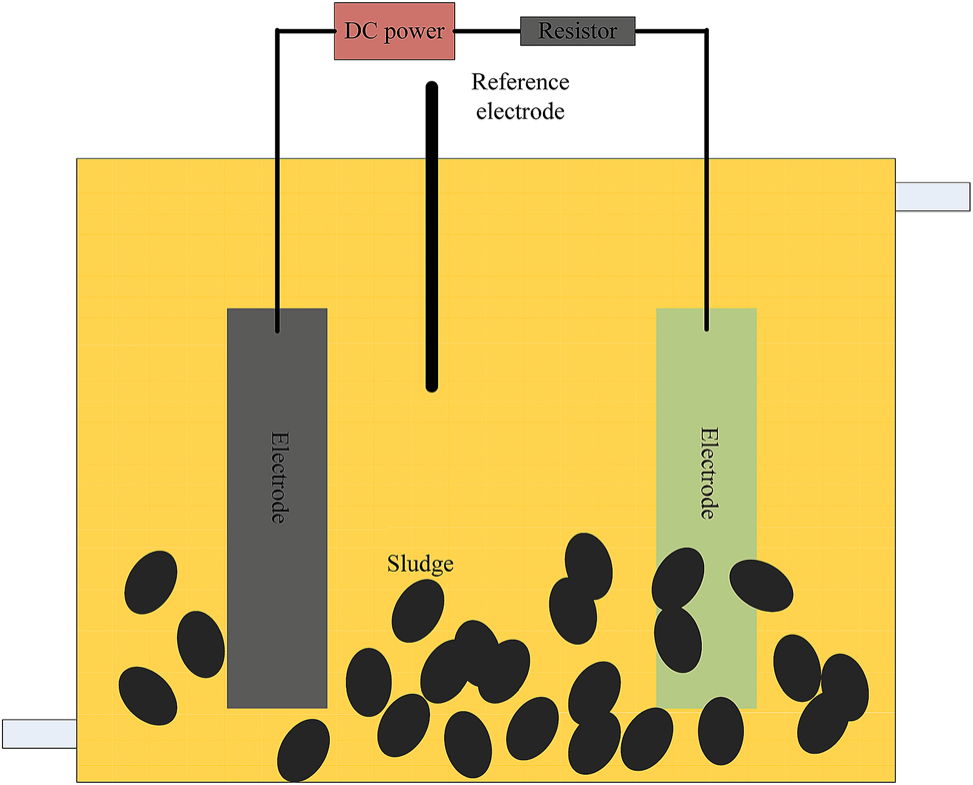
Schematic diagram of the reactor configuration.

The experiments were conducted in single-chambered microbial electrolysis fuels (BES) with a volume of 480 mL (6 × 8 × 10 cm) in batch assays (Fig. 1). A pair of graphite felt electrodes (Beijing Sanye Carbon Co., China) was used and the size was set according to the *V*/*S* (volume of the BES to the size of the electrode). A 1.5 V external electric field was added with a direct current power source (Victory3003D, China). A 10 Ω resistor was used in the circuit.

### 2.2 Synthetic wastewater

Synthetic wastewater was used in this study and the composition is described in our previous study.^1^ 2,4-Dichloronitrobenzene (DClNB) was used as the target pollutant and an initial dose of 50 mg L^−1^ was used in the assays. The BESs were inoculated with sludge taken from a steadily operated upflow anaerobic sludge blanket (UASB) in the lab.

### 2.3 Analytical method

DClNB and Cl^−^ were measured by high-performance liquid chromatography (HPLC) (Waters 2487, USA) and Cl^−^ was monitored by ion chromatography (IC) (Dionex 1100, USA) according to Chen *et al.*^1^

The fluorescence staining technique and confocal laser scanning microscopy (CLSM) (ZEISS, LSM710 NLO, Germany) were used to observe the distribution of live and dead cells. A LIVE/DEAD BacLight Bacterial Viability Kit (Invitrogen, CA, USA) was dissolved in 5 mL of sterile deionized water and mixed with equal bacterial suspension. The sample was placed under dark conditions for 15 min and observed by CLSM.

Electrochemical impedance spectroscopy (EIS) was also conducted on an electrochemical workstation to analyze the resistance of the reactor. A two-electrode system was used to measure the resistance of the whole reactor. The anode was used as the working electrode and the cathode was used as the counter electrode and reference electrode. The testing frequency ranged from 10^−2^ to 10^5^ Hz with an amplitude of 5 mV.

### 2.4 Experimental design

For the response surface models, the independent variables were *V*/*S* (*X*_1_), *D* (*X*_2_) and *P* (*X*_3_) and −1, 0 and +1 represented the low, center and high level of each variable. The DClNB removal rate (*Y*_1_) and ΔCl^−^ (*Y*_2_) were the dependent variables. The design and results of the experiments are presented in Table 1. The significance of the coefficient of the models was determined using *p*-values and the response variables were considered to be significant when *p* was below 0.05.

**Table 1:** Summary of the independent and dependent variables

Run	*X*_1_ a (*V*/*S*)	*X*_2_: *D*^b^ (cm)	*X*_3_: *P*^c^	*Y*_1_: removal rate mg L^−1^ h^−1^	*Y*_2_ (ΔCl^−1^) mg L^−1^
1	40	3	0.5	1.885	11.721
2	20	2	1	1.569	9.513
3	30	2	0.5	1.969	12.494
4	40	1	0.5	1.775	11.353
5	30	2	0.5	1.952	12.728
6	30	1	1	1.533	9.255
7	20	1	0.5	1.863	11.935
8	20	3	0.5	1.623	9.789
9	40	2	0	1.646	11.095
10	40	2	1	1.616	9.860
11	30	2	0.5	1.919	12.788
12	20	2	0	1.677	10.286
13	30	1	0	1.733	10.654
14	30	3	1	1.494	9.145
15	30	3	0	1.722	10.703

a Volume of the MEC/size of the electrode.

b Distance between the electrodes.

c Position of the electrode.

## 3 Results and discussion

### 3.1 Overview of the response models

Fitting of empirical models to the experimental data was conducted by RSM to describe the characteristics of the response. The mathematical–statistical relationship between the independent variables (*X*) and the response function (*Y*) is as follows:

1 *Y* = *b*_0_ + *b*_1_*X*_1_ + *b*_2_*X*_2_ + *b*_3_*X*_3_ + *b*_12_*X*_12_ + *b*_13_*X*_13_ + *b*_23_*X*_23_ + *b*_11_*X*_1_^2^ + *b*_22_*X*_2_^2^ + *b*_33_*X*_3_^2^

where *X*_1_, *X*_2_ and *X*_3_ represent the *V*/*S* ratio, interval and position, respectively.

Eqn (2) and (3) describe the response functions for ΔCl^−^ and DClNB removal rate.

2 *Y*_ΔCl^−^_ = 5.34962 + 0.28355*X*_1_ + 1.36388*X*_2_ + 7.09425*X*_3_ + 0.06285*X*_12_ − 0.0231*X*_13_ − 0.0795*X*_23_ − 6.10625 × 10^−3^*X*_1_^2^ − 0.85988*X*_2_^2^ − 7.4835*X*_3_^2^ (*R*^2^ = 0.9757)

3 *Y*_removal rate_ = 1.293 + 0.02902*X*_1_ + 0.05533*X*_2_ + 0.74083*X*_3_ + 8.75 × 10^−3^*X*_12_ + 3.9 × 10^−3^*X*_13_ − 0.014*X*_23_ − 7.68333 × 10^−4^*X*_1_^2^ − 0.08333*X*_2_^2^ − 0.97133*X*_3_^2^ (*R*^2^ = 0.9538)

The closer the correlation coefficient (*R*^2^) is to 1, the more accurate the polynomial equation will be.^20^ The calculated *R*^2^ (0.9757 and 0.9538) indicated that the predictions of the response function were in line with the experimental one at the confidence level of 95%. The absolute value of the coefficient of *X*_2_ is significantly higher than that of the other variables, indicating that the proportion of the electrodes immerged in the sludge is the main factor controlling the ΔCl^−^ and DClNB removal rate.

The variance analyses (ANOVA) in Tables 2 and 3 describe the fitting results for the response surface model. The significance of the model is judged by the *F*-value and *p*-value. The *F*-value represents the ratio of regression mean square to the estimated parameter standard deviation, while the *p*-value is the probability of the occurrence of the *F*-value.^21^ Both models are significant in this study (*p*-values are 0.0076 and 0.0016). The results indicate that the terms *X*_3_ and *X*_3_^2^ are significant with *p*-values below 0.05, indicating that the position of the electrodes is the most important factor affecting the DClNB removal rate and ΔCl^−^. The results are in agreement with those of the coefficient analyses.

**Table 2:** ANOVA test for response function *Y*_removal rate_^a^

Source	Sum of squares	df	Mean square	*F* value	*p*-value, prob > *F*	
Model	0.32	9	0.036	11.48	0.0076	Significant
*A-V*/*S*	4.513 × 10^−3^	1	4.513 × 10^−3^	1.43	0.2847	
*B-D*	4.050 × 10^−3^	1	4.050 × 10^−3^	1.29	0.3097	
*C-P*	0.040	1	0.040	12.73	0.0161	
*AB*	0.031	1	0.031	9.74	0.0262	
*AC*	1.521 × 10^−3^	1	1.521 × 10^−3^	0.48	0.5178	
*BC*	1.960 × 10^−4^	1	1.960 × 10^−4^	0.062	0.8128	
*A* ^2^	0.022	1	0.022	6.93	0.0464	
*B* ^2^	0.026	1	0.026	8.15	0.0356	
*C* ^2^	0.22	1	0.22	69.23	0.0004	
Residual	0.016	5	3.145 × 10^−3^			
Lack of fit	0.014	3	4.811 × 10^−3^	7.44	0.1207	Not significant
Pure error	1.293 × 10^−3^	2	6.463 × 10^−4^			
Cor total	0.34	14				

a *R* ^2^ = 0.9538; Adj *R*^2^ = 0.8707; Pred *R*^2^ = 0.3135.

**Table 3:** ANOVA test for response function *Y*_ΔCl^−^_a

Source	Sum of squares	df	Mean square	*F* value	*p*-value, prob > *F*	
Model	21.4	9	2.38	22.26	0.0016	Significant
*A-V*/*S*	0.79	1	0.79	7.35	0.0422	
*B-D*	0.42	1	0.42	3.96	0.1033	
*C-P*	3.08	1	3.08	28.85	0.0030	
*AB*	1.58	1	1.58	14.79	0.0120	
*AC*	0.053	1	0.053	0.50	0.5113	
*BC*	6.320 × 10^−3^	1	6.320 × 10^−3^	0.059	0.8175	
*A* ^2^	1.38	1	1.38	12.89	0.0157	
*B* ^2^	2.73	1	2.73	25.56	0.0039	
*C* ^2^	12.92	1	12.92	121.00	0.0001	
Residual	0.53	5	0.11			
Lack of fit	0.49	3	0.16	6.71	0.1324	Not significant
Pure error	0.048	2	0.024			
Cor total	21.94	14				

a *R* ^2^ = 0.9757; Adj *R*^2^ = 0.9318; Pred *R*^2^ = 0.6407.

**Fig. 2:**
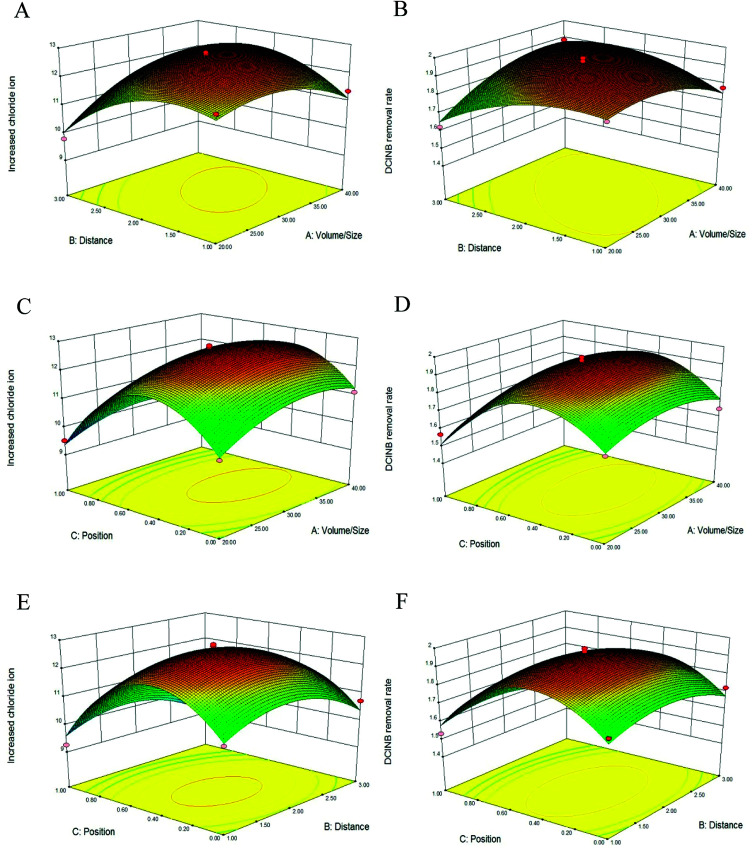
Three-dimensional response surface plots. Effect of volume/size ratio and distance on ΔCl^−^ (A) and DClNB removal rate (B); effect of volume/size ratio and position on ΔCl^−^ (C) and DClNB removal rate (D); effect of distance and position on ΔCl^−^ (E) and DClNB removal rate (F).

The three-dimensional (3D) response surface plots are presented in Fig. 2. The interaction effects of two variables on the response functions are revealed in these plots. Fig. 2A and B describe the interaction of the *V*/*S* ratio with the interval when 50% of the electrodes are immerged in the sludge. Fig. 2C and 2D represent the interaction of the *V*/*S* ratio with the electrode position when the interval between the electrodes is 2 cm. Fig. 1E and F represent the interaction of the electrode position with the interval when the *V*/*S* ratio is at the center point of 30. Each plot exhibits an obvious peak, indicating that the optimal point was well concluded as inside the design boundary.^22^ It has been reported that the contour plots reflect the strength of the interaction between the variables. The interaction can be ignored if the contour lines are close to a circle. On the contrary, the interaction is strong if the contour lines look like ellipses.^23^ As depicted in Fig. 2A and B, the contour lines are close to circles, indicating that the interaction between the interval and *V*/*S* ratio can be ignored. The contour lines in Fig. 2C–F are close to ellipses, indicating that the interactions between the position and *V*/*S* ratio, and the position and interval were strong.

### 3.2 Validation of the regression model

In order to achieve the maximum DClNB removal rate and ΔCl^−^, the optimum parameters were used according to the RSM. The *V*/*S* ratio, interval and position were 31.75, 1.95 cm and 42%, respectively. The experiment was conducted in triplicate. The results indicated that the DClNB removal rate and ΔCl^−^ were 1.819 ± 0.037 mg L^−1^ h^−1^ and 11.894 ± 0.180 mg L^−1^, respectively. The deviations from the predicted values were both below 5%, indicating that the regression was applicable for predicting the DClNB removal rate and ΔCl^−^.

### 3.3 The effect of electrode position on reactor performance

According to the results above, electrode position was the key factor influencing reactor performance. Therefore, a continuous experiment was conducted with three BESs. The electrodes of the BESs were immerged in the sludge 0%, 50% and 100%, while the interval between the electrodes and the *V*/*S* were 2 cm and 40. The COD and DClNB concentration were maintained at 500 and 100 mg L^−1^. The reactor performances were compared from the perspectives of current, pollutant transformation, EIS, *etc.*

#### 3.3.1 Differences in DClNB transformation

**Fig. 3:**
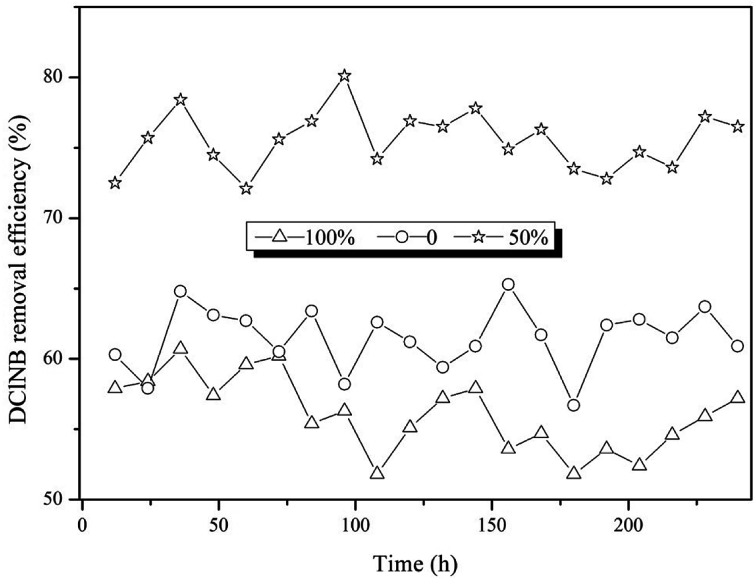
The effect of electrode position on pollutant removal.

DClNB transformation highly depended on the electrode position (Fig. 3). The DClNB removal efficiencies in the 0%, 50% and 100% immerged reactors were 56.1 ± 2.7%, 75.5 ± 2.1% and 61.5 ± 2.2%, respectively. The 50% immerged electrodes had the best performance, followed by the 0 and 100% immerged ones. The results indicated that having an appropriate proportion of the electrodes immerged in the sludge could effectively improve pollutant transformation. Kong *et al.* reported that in a reactor with 1/4 soaking electrode, the functional microbes in the sludge could migrate to the upper part of the electrode more easily, contributing to the formation of a biofilm on the electrode surface.^24^ This might be related to the electron transfer through the electrode. However, the biofilms on the over-immerged electrodes, which might not be bio-electrocatalytically active, would exhibit electron transfer resistance. Moreover, large amounts of biomass that could restrict the mass transfer process might be developed.^25^

#### 3.3.2 Differences in current

**Fig. 4:**
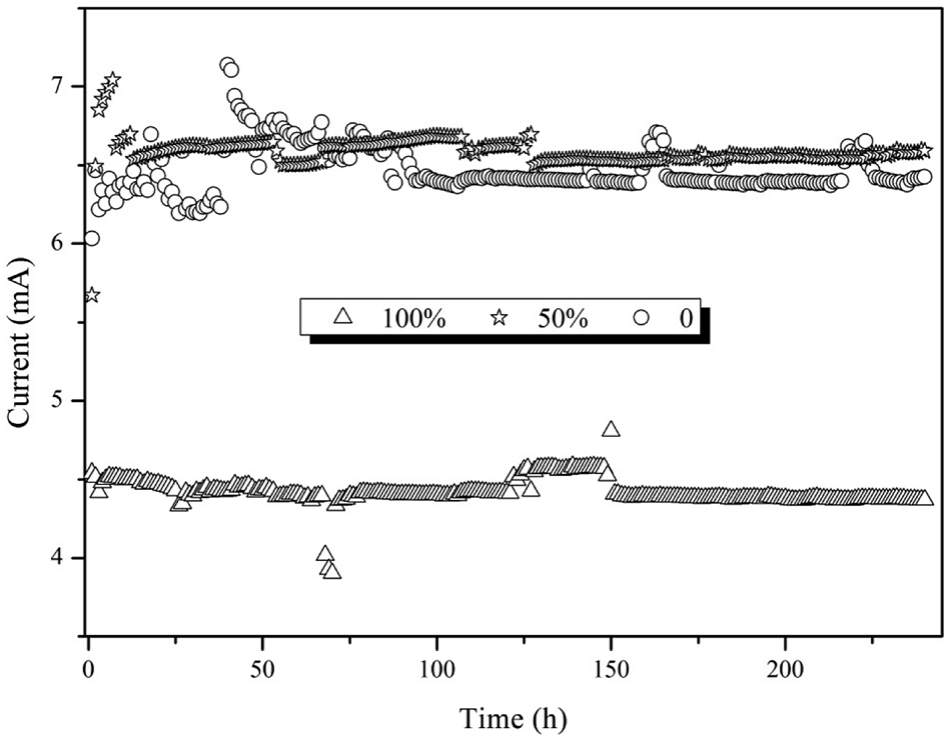
The effect of electrode position on the current.

**Fig. 5:**
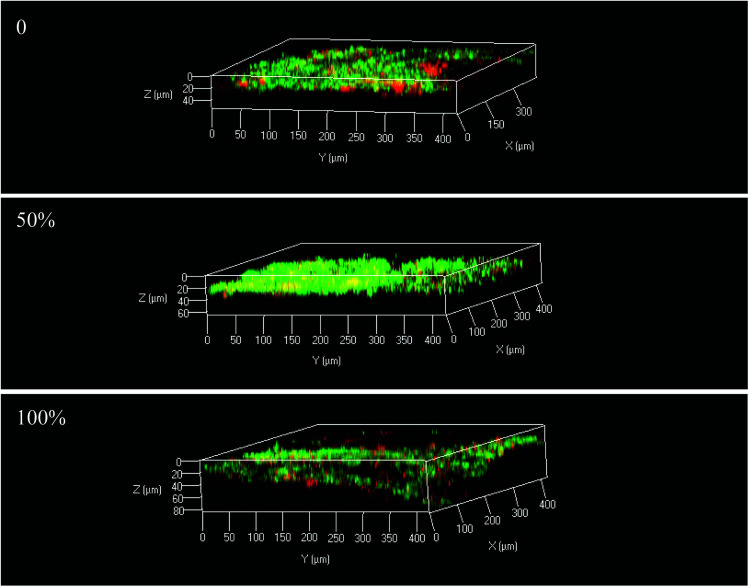
CLSM graphs of the electrode biofilms.

As discussed above, the thickness of the biofilm might lead to differences in electron transfer between the electrodes and electron acceptors. Fig. 4 reveals the current in the 0%, 50% and 100% immerged reactors (6.47 ± 0.15, 6.59 ± 0.09 and 4.42 ± 0.08 mA). The highest current was observed for the 50% immerged reactor, which was 1.5-fold that of the 100% immerged reactor. This might be due to the fact that the microbes attached to the 50% immerged electrode had higher microbial activity, leading to the evolution of the microbial community and diversity.^26^ The current generation in the BES was reported to be influenced by the transfer of protons, substrate and metabolites between the solution and electrodes.^27^ Hence, the differences in electrode position might result in different transfer capacities, leading to differences in current generation. The microbes in the 50% immerged electrode might have higher electroactivity, which would be beneficial to the electron transfer between the electrodes and microbes, resulting in higher current generation. Michie *et al.* reported that mass transfer and biocatalytic reactions would be inhibited with over-thick biofilms.^25^ Therefore, the biofilms were observed by confocal laser scanning microscopy (CLSM) to reveal if there was any difference in biofilm characteristics (Fig. 5). The CLSM graphs indicated that most dead microbes were located in the inner layer of the biofilm, while living microbes were located in the outer layer. It was found in Fig. 4 that the 100% immerged reactor had the thickest biofilm and this was in accordance with the results above, *i.e.*, the over-thick biofilm reduced the current density and pollutant removal efficiency.

#### 3.3.3 Differences in EIS

**Fig. 6:**
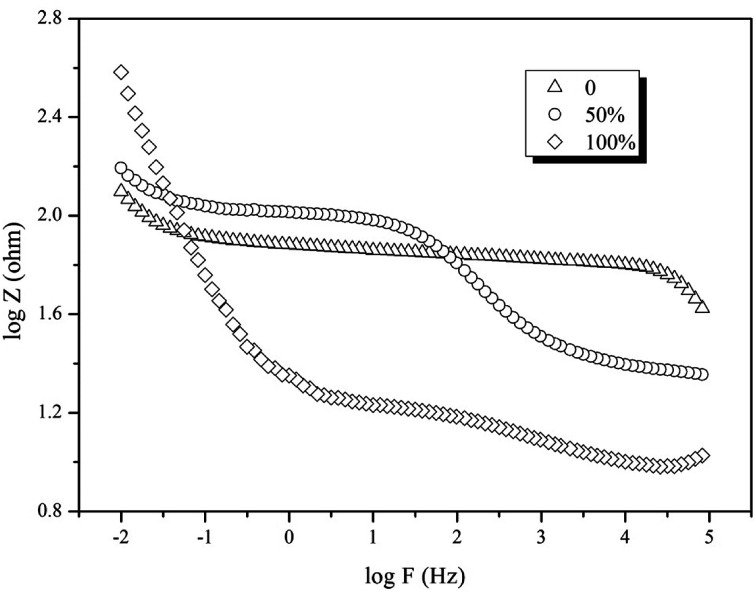
Effect of electrode position on the electrochemical characteristics.

EIS was conducted and a Bode graph was used to describe the relationship between the resistance and frequency. Fig. 6 indicates that the resistance of the 100% immerged reactor is much higher that of the other ones (398.1 Ω *vs.* 134.9 Ω and 158.5 Ω). The low-frequency region represents the resistance in charge transfer and the higher the value is, the slower the charge transfer. A higher value in this region can be attributed to slower kinetics of charge transfer reactions associated with the redox process,^28^ confirming that the 100% immerged electrode had higher impedance than the other electrodes. This indicated that when the electrode was placed in bulk solution (0% immerged) or 1/2 part in the sludge (50% immerged), the electrochemical reaction would be accelerated for efficient electron transfer. However, when the electrode was totally immerged in the sludge, microbes would attach on the surface of the electrode to form a thick biofilm, reducing the effective contact area of the electrode with the pollutant, and leading to a decrease in pollutant transformation.^24^

Taking the results above together, electrode position influences the formation of the biofilm, leading to differences in resistance, current and pollutant removal efficiency. The over-thick biofilm in the 100% reactor would inhibit pollutant transformation. Hence, the proportion of the electrode immerged in the sludge should be further investigated in future research.

## 4 Conclusions

Response surface methodology (RSM) was applied to optimize the operational conditions and the optimum conditions for the *V*/*S* ratio, interval and position were 40, 2.31 cm and 0.42. The pollutant removal rate and increased Cl^−^ achieved under these conditions were close to the predicted ones, indicating the feasibility of the model for the prediction of DClNB transformation in the BES. DClNB transformation was inhibited when the electrodes were completely immerged in the sludge due to over-thick biofilms. Specifically, the resistance increased when the electrodes were completely immerged in the sludge, leading to a decrease in the current and pollutant removal efficiency. The current study confirms the feasibility of RSM for the optimization of DClNB transformation in a lab-scale BES, but more scaled-up studies should be conducted in the future.

## Conflicts of interest

There are no conflicts to declare.

## Supplementary Material
